# Utilization of Traditional Korean Medicine Services by the Older Population: A Cross-Sectional Study

**DOI:** 10.3390/healthcare10081444

**Published:** 2022-08-01

**Authors:** Angela Dong-Min Sung, You-Sang Baik, Soobin Jang, Jang-Kyung Park, Hyun-Kyung Sung, Ji-Yeon Lee, Byung-Cheul Shin, Sung-Yong Choi, Soo-Hyun Sung

**Affiliations:** 1 Department of Policy Development, National Development Institute of Korean Medicine, Seoul 04554, Korea; angelasung84@nikom.or.kr (A.D.-M.S.); baikys@nikom.or.kr (Y.-S.B.); 2 Department of Korean Medicine Classics, College of Korean Medicine, Kyung Hee University, Seoul 02453, Korea; 3 Department of Preventive Medicine, College of Korean Medicine, Daegu Haany University, Gyeongsangbuk-do, Gyeongsan 38609, Korea; suebin@nate.com; 4 Division of Clinical Medicine, School of Korean Medicine, Pusan National University, Yangsan 50612, Korea; vivat314@pusan.ac.kr (J.-K.P.); drshinbc@pusan.ac.kr (B.-C.S.); 5 Department of Korean Medicine Pediatrics, School of Korean Medicine, Semyung University, Jecheon 27136, Korea; shksolar@gmail.com; 6 Department of Obstetrics and Gynecology, Daejeon Korean medicine Hospital of Daejeon University, Daejeon 35235, Korea; jyounl@daum.net; 7 Seoul Metropolitan Government Big Data Division, Official, Seoul 04524, Korea

**Keywords:** traditional Korean medicine (TKM), complementary and alternative medicine (CAM), national survey TKM usage, older adults, medical service utilization

## Abstract

Korean healthcare service is divided into conventional medicine and traditional Korean medicine (TKM). This cross-sectional study compared the older population (65 years and older) with two groups of the general population (19–50 years and 51–64 years) to understand the current patterns in the use of TKM services by the older population. Using data from the 2017 National Survey of TKM Usage, we analyzed the main purpose and diseases or symptoms for TKM use, the reason for choosing TKM over other types of medicine, and the treatments provided. Both age groups sought TKM services to “[treat] a disease”. The top five diseases and symptoms in the older adult (65 and older) group were musculoskeletal and are as follows: arthritis and joint diseases (37.8%, *n* = 166), disc-related diseases (25.5%, *n* = 112), back pain (8.4%, *n* = 37), frozen shoulder and shoulder pain (7.7%, *n* = 34), and sprain (5.9%, *n* = 26). The most frequently used treatments for these diseases were acupuncture, moxibustion, cupping, and physiotherapy. The present study suggests that older adults primarily used TKM clinics for the treatment of musculoskeletal diseases. Further studies are necessary to assess the clinical effectiveness of conventional medicine versus TKM versus a combination of both in treating musculoskeletal disorders.

## 1. Introduction

In many regions of the world, life expectancy has increased and fertility rates have declined, leading to simultaneous shrinking and aging in populations [1,2,3]. Globally, people over the age of 65 years are the fastest-growing segment of the population, and in 2019—for the first time in human history—they outnumbered children younger than 5 years of age [1,2]. In Korea, growth rate of the older population is the fastest in the world. The proportion of the older population in the country was 7.2% in 2000 and 11% in 2010. It is expected to reach 20.8% by 2026 and 34% by 2050 [4,5]. The aging population presents a worldwide challenge that is gaining traction as an important topic in national government policies.

Population aging means an increased demand in medical services for the older population [6]. Healthcare usage and expenditure among older adults are higher than those of the younger population and continue to grow rapidly [7]. Due to the diverse treatment approaches used to treat the older adults, it is necessary to use a systematic approach that can meet their needs [7]. The subjective health status of older adults warrants more attention from policymakers and researchers because this population has a variety of chronic conditions that lead to their poor health status [8,9].

Changing disease patterns and an increase in the need for chronic disease management have increased the interest in the use of traditional, complementary, and alternative medicine (T&CM) across the globe [10]. When compared to younger adults, the older population prefers using T&CM as an alternative to conventional treatment [11,12,13,14]. The higher prevalence of multiple medical conditions, decreased functional activity, assumption of T&CM safety compared to conventional medicine safety, and perceived therapeutic effects are among the main reasons why T&CM utilization appears to be more prevalent in older adults [13,14].

Korea has a dual medical system, and traditional Korean medicine (TKM) is included in the system along with conventional medicine [15]. In other words, Korean healthcare service is divided into conventional medicine and TKM. Of the available TKM treatments, acupuncture, moxibustion, cupping, and manual therapies (e.g., chuna) are covered by medical insurance [15]. Despite the widespread availability of TKM, the prevalence of TKM use in the older adults has not yet been investigated on a national level. Statistics Korea has undertaken an assessment of TKM usage as a certified national statistic every 3 years since 2008 [16]. This assessment covers all household members aged 19 years and older, thus providing a representative sample of the country [17].

In this study, we comparatively analyzed the older population (65 years and older) and the general population (19–50 years and 51–64 years) in Korea to understand the current patterns and characteristics of TKM use by the older population. By doing so, we hope to contribute to the development of T&CM policies for the older adults in a country where traditional medicine is well established.

## 2. Literature Review

Schnabel et al. [12] investigate the current level of use, opinions, and preferences of CAM were investigated for 400 German older people over 70 years old. In particular, 33.3% of the respondents used herbal remedies. Two-thirds of participants (64.9%) generally preferred the combination of CAM and conventional medicine. Ayele et al. [14] conducted an institution-based quantitative cross-sectional survey of elderly patients with chronic diseases in Ethiopia to evaluate the prevalence and reason for the use of CAM. As a result, 240 people—or 74% of all respondents—reported using CAM, and herbal medicine treatment was the most commonly used at 54%. Sung et al. [18] conducted a survey of 16 local governments to investigate the status of community medical services for TKM for the older people and to raise awareness of the current opinions and services of TKM institutions. As a result, 11 out of 16 local governments (68.8%) provided TKM home care services. A total of 136 TKM clinics provided home care services to 598 older people with musculoskeletal disorders. As a result, pain and quality of life were significantly improved after TKM service. Morrissey et al. [19] examined seven cross-sectional data from European social surveys in 21 countries for participants aged 55 and older who reported musculoskeletal pain that interfered with daily activities for 12 months to examine the use and characteristics of CAM. A total of 1657 people (33.5%) reported using at least one CAM treatment in the previous year, and the rate of reporting herbal remedies was popular at 5.3%.

## 3. Materials and Methods

### 3.1. Data Sources

The data source of this study was the 2017 National Survey for Usage and Consumption of Traditional Korean Medicine (National Approved Statistics Number: 117087). This study was conducted using microdata approved by the National Institute of Korean Medicine Development (no. 2020-03). The National Survey for Usage and Consumption of Traditional Korean Medicine has been conducted four times since 2008, after every three years, in order to secure basic data for enhancing accessibility to TKM, upgrading national policies, and determining policy priorities in accordance with the demands of T&CM services among the people of Korea. The first survey, in 2008, focused om TKM usage. This was followed by a survey on the consumption of traditional herbal medicine (first survey) in 2009. In 2011, the two separate surveys were integrated into one survey on TKM usage. Since then, the survey has been conducted every 3 years.

The 2017 survey was conducted by the National Development Institute of Korean Medicine (NIKOM) in conjunction with Gallup. In this report, a total of 6914 participants were questioned regarding their awareness of and experience with T&CM. This group included 5000 male and female members of the general public who were 19 years of age and older, along with 1010 outpatients and 904 inpatients of TKM clinics [17]. Among all the data collected over the years, we used the data on the usage of TKM obtained during the fourth survey that took place in 2017.

### 3.2. Sample Selection

**Figure 1:**
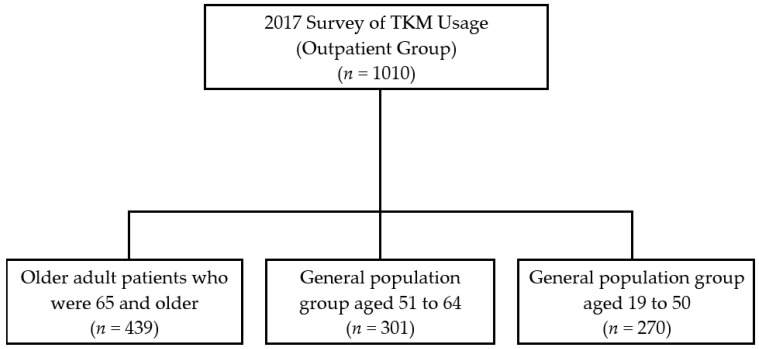
Flow chart of inclusion and exclusion of participants from the 2017 National Survey of TKM Usage. TKM: Traditional Korean Medicine.

The data compared and analyzed in this study were from a subset of the 1010 outpatients in the 2017 National Survey for Usage and Consumption of Traditional Korean Medicine. For our study, we excluded 301 patients who were 51–64 years old and only included 439 older patients (65 years or older) along with 301 patients who were 51–64 and 270 patients who were 19–50 years old, amounting to 709 participants in total. In this study, the older group (65 years and older) was compared with the young adult group (19–50 years) and the adult group (51–64 years). The selection process of the participants is shown in Figure 1.

### 3.3. Analysis Items

The items analyzed included demographics (sex, marital status, education, occupation, household income, perceived health, and health security types), the purpose of using TKM, reason for choosing TKM over traditional medicine, key symptoms and diseases for which TKM was used, and treatment methods used for each symptom and disease. Among the questionnaires, the questionnaire items used for analysis were added as Additional File 1 in Supplementary Materials. All responses were made only to patients who had used TKM for the past year. 

### 3.4. Statistical Analysis

To better understand the general characteristics of the participants included in the analysis, frequencies and ratios were calculated. The correlations between the variables were analyzed using the χ^2^-test. In addition, the key treatment methods for each symptom in the older adult group were analyzed and summarized in a chart. Statistical analyses were conducted using IBM SPSS Statistics for Windows, version 25 (IBM Corp., Armonk, NY, USA), and the level of significance was set at 5% (*p* < 0.05). 

## 4. Results

### 4.1. Characteristics of the Older Adult Group (Ages 65 and Older) and General Population Group (Ages 19–50 and 51–64)

The demographics of the outpatients using TKM services are shown in Table 1. Of the outpatients, 43.5% (*n* = 439) were 65 years of age and older, while 26.7% (*n* = 270) were 19–50 years of age and 29.8% (*n* = 301) were 51–64 years of age. As for the sex distribution among the older adult group, 32.8% (*n* = 144) were male while 67.2% (*n* = 295) (*p* = 0.014) were female. Regarding the education level, 60.6% (*n* = 266) of the older adult group had completed elementary school education or lower, while 40.4% (*n* = 109) of the general population group aged 19–50 had attained university education and of the general population group aged 51–64 had completed middle school education (*p* < 0.0001). As for employment, those in the older adult group who answered that they were unemployed accounted for 67.9% (*n* = 298), thus outnumbering those who were employed (32.1%, *n* = 298). In the general population group aged 19–50, 75.9% (*n* = 205) were employed, outnumbering those who were unemployed (24.1%, *n* = 65); and in the general population group aged 51–64, 70.1% (*n* = 211) were employed, outnumbering those who were unemployed (29.9%, *n* = 90) (*p* < 0.0001). Regarding income, 44.4% (*n* = 195) of the older adult group reported an income ranging from 1500 United States dollar (USD) to less than 3000 USD, while 37.4% (*n* = 101) of the general population aged 19–50 and 38.9% (*n* = 117) of the general population aged 51–64 reported an income from 3000 USD to less than 4500 USD (*p* < 0.0001). Regarding the perceived health status, 33.7% (*n* = 148) of the older adult group and 73.3% (*n* = 198) of the general population group believed that they were in good health (*p* < 0.0001). Regarding the types of health insurance, 62.9% (*n* = 276) of the older adult group was covered by the national resident health insurance, while the general population group aged 19–50 and the general population aged 51–64 were primarily covered (74.1%, *n* = 200 and 64.5%, *n* = 194) by the national occupational health insurance (*p* < 0.0001).

### 4.2. Demographics of TKM Use in Outpatients

The purposes for which outpatients utilized TKM services are shown in Table 2. “Treating a disease” was the most common answer among the older adult (96.3%, *n* = 423), general population groups aged 19–50 (81.5%, *n* = 220) and general population groups aged 51–64 (90.0%, *n* = 271).

The reasons for choosing TKM services for outpatients are listed in Table 2. According to the survey, 65.6% (*n* = 288) of the older adult group answered that they chose TKM because it was highly effective, followed by those who preferred it because the clinic was in close proximity (10.7%, *n* = 47), followed by the perception of fewer side effects (9.6%, *n* = 42), and specialized treatment options (3.9%, *n* = 17). The reason why the general population group aged 51–64 chose TKM was similar to the older adult group. In the general population group aged 19–50, 62.2% (*n* = 168) answered that they chose TKM because it was highly effective, followed by the perception of fewer side effects (11.9%, *n* = 32), the clinic being in close proximity (8.9%, *n* = 24), and the reputation of the clinic or a personal recommendation (6.7%, *n* = 18).

### 4.3. The Main Symptoms of Outpatient Groups Using TKM Services

The top five symptoms and diseases for which older outpatients used TKM services are shown in Table 3 and as follows: osteoarthritis, 37.8% (*n* = 166); disc-related disease, 25.5% (*n* = 112); back pain, 8.4% (*n* = 37); frozen shoulder and shoulder pain, 7.7% (*n* = 34); and sprain, 5.9% (*n* = 26). The top five symptoms and diseases for which the general population outpatients aged 51–64 used TKM services are shown in Table 3 and as follows: sprain, 27.6% (*n* = 83); osteoarthritis, 24.6% (*n* = 74); back pain, 11.0% (*n* = 33); frozen shoulder and shoulder pain, 9.6% (*n* = 29); disc related disease, 8.3% (*n* = 25). The top five symptoms and diseases for which the general population outpatients aged 19–50 used TKM were Table 3: disc-related disease, 28.9% (*n* = 78); osteoarthritis, 18.9% (*n* = 51); back pain, 11.1% (*n* = 30); sprain, 9.6% (*n* = 26); and frozen shoulder and shoulder pain, 8.5% (*n* = 23).

### 4.4. Treatments Used for the Top Five Symptoms in the Older Outpatient Group

Table 4 shows the distribution of treatments used for the top five symptoms in the older outpatient group. The most frequently used treatments for the top five diseases were acupuncture, moxibustion, cupping, and physiotherapy.

## 5. Discussion

In this cross-sectional study, we used national data to compare the three population groups, the older adult group (65 years and older) and the general population group aged 19–50 years and the general population group aged 51–64 years, to understand the patterns and characteristics of the use of TKM by the older adults. We sought to examine the purpose of TKM use in older adults, their reason for choosing TKM over traditional medicine, and treatments for the diseases, in order to find a point of policy intervention.

The general population used TKM clinics to treat diseases, improve their overall health, receive treatment for car accident injuries, and for other purposes. In contrast, the majority of the older adults (96.3%) used TKM clinics to treat diseases. More than half of this population used these clinics to treat musculoskeletal diseases, such as arthritis and joint diseases (37.8%), and spinal diseases (25.5%). This is not surprising, as musculoskeletal diseases are common among older adults [20].

The top three reasons for the older adult and general population groups to choose TKM over traditional medicine were the treatment options, proximity, and perception of decreased side effects. TKM can be used as an alternative treatment for patients that are difficult to treat with conventional medicine. In addition, part of its popularity is attributed to the perceived benefit of decreased side effects when compared to those in conventional medicine [21]. Hence, it is not surprising that many participants reported choosing TKM over other treatment modalities due to “good treatment effects” or “low side effects”. In addition, 10.7% of the older adult participants answered that they chose TKM because the clinics were closer to their residence. In older adults, walking is slow, unstable, and inefficient, the timing and coordination of stepping with their posture, and phases of gait are poor [22]. Hence, the older adults preferred TKM clinics because they were closer and more accessible from their residences. In fact, many countries are building community care systems, such as home care, customized for their specific situations [18]. Therefore, the preference for TKM clinics that are more accessible would be the basis for a national governmental policy to introduce TKM home care for older adults.

In addition to the older adult group, both of the general population groups also had the same top five purposes for using TKM for all musculoskeletal diseases. Musculoskeletal diseases are the main category of diseases for which TKM is used, thus supporting the findings of previous studies [23,24]. As the older adult population increases worldwide, the morbidity rate of musculoskeletal diseases is expected to increase even further. Therefore, it is necessary to develop policies that can enhance maritime accessibility and care for musculoskeletal diseases in the older population. In addition, it is necessary to empirically verify whether (1) singular TKM treatments are effective, or (2) the combined treatment of TKM and conventional medicine is effective, through randomized controlled trials that compare and analyze the treatment of musculoskeletal diseases.

The five most frequently used TKM treatments by the older adults for the diseases discussed were acupuncture, moxibustion, cupping, and physiotherapy. Other treatments (e.g., pharmacopuncture, herbal extract, herbal decoction, or chuna therapy) were used in low frequency. Korea has a dual medical system that includes both TKM and conventional medicine. TKM treatments such as acupuncture, moxibustion, cupping, and physical therapy are covered by national insurance, which may account for their higher frequency of use [25]. However, chuna was less frequently used even though it is covered by the national health insurance. This can be explained by the fact that chuna has only been reimbursed by the national health insurance since 2019, while the results of this survey were based on data from a survey conducted in 2017. In the future, supportive policies will be necessary to provide healthcare coverage for certain treatment modalities, such as pharmacopuncture, herbal extracts, and herbal decoctions.

There are some limitations to our cross-sectional study. First, this study did not apply the same methodology as the regression model, so it was not possible to identify which factors affect use of TKM services by older adults. Therefore, further studies are needed, and we hope that various studies will be conducted to identify factors for the use of TKM services for the disabled, women, and patients with specific symptoms/disorders. Second, the study divided the frequent diseases and symptoms treated by TKM into general categories of arthritis and joint diseases, spinal diseases, back pain, frozen shoulder and shoulder pain, sprain, strain, and tension, making it difficult to identify the specific diseases. The difference between spinal diseases and back pain remains unclear. Therefore, in future studies, it would be beneficial to identify the specific names of diseases for each category, which will help to develop optimized policies based on the usage and preferences of TKM for specific diseases. Third, this study covered the use of TKM by the older population of Korea, and is therefore difficult to generalize for other countries. This is because the types of interventions, institutions, practitioners, policies, and systems differ worldwide.

This research describes the first survey using samples that are representative of the national population in Korea. In order to develop policy in a country, national statistics and survey become important data, and overseas cases serve as reference data. In this aspect, this study will contribute as follows: (1) Use as evidence for developing national policy for the older population in Korea; (2) Use as a reference for developing T&CM policies for older adults in a country where traditional medicine is well established.

## 6. Conclusions

In conclusion, this study compared the older adult group (65 years and older) and the two group of general population groups (ages 19–50 and 51–64) to identify the current pattern of TKM use amongst the older population by analyzing the 2017 national survey of TKM usage. The results suggest that older adults use TKM clinics for the purpose of getting treatment for their musculoskeletal diseases, and the treatments of choice were those covered by the national health insurance (e.g., acupuncture, moxibustion, cupping, and physiotherapy). In the future, it will be necessary to consider the results of this study when establishing policies for providing TKM services for the older population in Korea. In addition, a comparative analysis between conventional medicine, TKM, and a combination of both to treat musculoskeletal disorders is recommended.

With the continued proportional increase in the older population, it is now time to establish a new medical social welfare system and policies that include the expansion of the national health insurance coverage for the older population, especially with regard to musculoskeletal diseases, along with the introduction of a family doctor system.

## Figures and Tables

**Table 1:** Information and characteristics of the older adult group (65 years and older) and general population group (19–50 years).

Category		65 and Older *n* (%)	51 to 64 *n* (%)	19 to 50 *n* (%)	Total *n* (%)	χ^2^ (*p*)
Total number		439 (43.5)	301 (29.8)	270 (26.7)	1001 (100.0)	-
Sex	Male	144 (32.8)	111 (36.9)	118 (43.7)	373 (36.9)	8.531 (*p* = 0.014)
	Female	295 (67.2)	190 (63.1)	152 (56.3)	637 (63.1)	
Marital status	Unmarried	0 (0.0)	4 (1.3)	86 (31.9)	90 (8.9)	239.329 (*p* < 0.0001)
	Married (bereaved, divorced, and common-law included)	439 (100.0)	297 (98.7)	184 (68.1)	920 (91.9)	
Academic background	Primary or lower school graduate	266 (60.6)	13 (4.3)	2 (0.7)	281 (27.8)	571.386 (*p* < 0.0001)
	Middle school graduate	142 (32.3)	171 (56.8)	68 (25.2)	381 (37.7)	
	High school graduate	19 (4.3)	39 (13.0)	91 (33.7)	149 (14.8)	
	University or higher school graduate	12 (2.7)	78 (25.9)	109 (40.4)	199 (19.7)	
Job	Yes	141 (32.1)	211 (70.1)	205 (75.9)	557 (55.1)	168.459 (*p* < 0.0001)
	No	298 (67.9)	90 (29.9)	65 (24.1)	453 (44.9)	
Household income	Less than 1500 USD	148 (33.7)	10 (3.3)	5 (1.9)	163 (16.1)	351.725 (*p* < 0.0001)
	1500 USD less than 3000 USD	195 (44.4)	64 (21.3)	50 (18.5)	309 (30.6)	
	3000 USD less than 4500 USD	62 (14.1)	117 (38.9)	101 (37.4)	280 (27.7)	
	4500 USD less than 6000 USD	19 (4.3)	61 (20.3)	75 (27.8)	155 (15.3)	
	No less than 6000 USD	15 (3.4)	49 (16.3)	39 (14.4)	103 (10.2)	
Health status	Good	148 (33.7)	190 (63.1)	198 (73.3)	536 (53.1)	136.856 (*p* < 0.0001)
	Bad	123 (28.0)	25 (8.3)	23 (8.5)	171 (16.9)	
	Average	168 (38.3)	86 (28.6)	49 (18.1)	303 (30.0)	
Medical security type	Health insurance (district insurance)	276 (62.9)	106 (35.2)	65 (24.1)	447 (44.3)	121.537 (*p* < 0.0001)
	Health insurance (workplace insurance)	156 (35.5)	194 (64.5)	200 (74.1)	550 (54.4)	
	Medical care	6 (1.4)	1 (0.3)	4 (1.5)	11 (1.1)	
	Miscellaneous	1 (0.2)	0 (0.0)	1 (0.4)	2 (0.2)	

TKM: Traditional Korean Medicine, USD: United States Dollar.

**Table 2:** Purpose of using TKM and reasons for choosing TKM over traditional medicine.

Category		65 and Older *n* (%)	51 to 64 *n* (%)	19 to 50 *n* (%)	Total *n* (%)	χ^2^ (*p*)
Purposes of using TKM	Treating a disease	423 (96.3)	271 (90.0)	220 (81.5)	914 (90.5)	51.809 (*p* < 0.0001)
	Health promotion	2 (0.5)	5 (1.7)	15 (5.5)	22 (2.2)	
	Cosmetic treatment	0 (0.0)	2 (0.7)	7 (2.6)	9 (0.9)	
	Treatment for car accident	14 (3.2)	23 (7.6)	28 (10.4)	65 (6.4)	
Reason for choosing TKM	High effective	288 (65.6)	194 (64.5)	168 (62.2)	650 (64.4)	18.116 (*p* = 0.020)
	Less side effects of surgery and examination	13 (3.0)	6 (2.0)	6 (2.2)	25 (2.5)	
	Less side effects	42 (9.6)	29 (9.6)	32 (11.9)	103 (10.2)	
	Low cost of treatment	11 (2.5)	3 (1.0)	1 (0.4)	15 (1.5)	
	to hear the detailed explanation	5 (1.1)	4 (1.3)	0 (0.0)	9 (0.9)	
	Treatment specific to the disease	17 (3.9)	13 (4.3)	20 (7.4)	50 (5.0)	
	Clinic’s being closer	47 (10.7)	39 (13.0)	24 (8.9)	110 (10.8)	
	Good facilities	2 (0.5)	0 (0.0)	1 (0.4)	3 (0.3)	
	Famous and recommendation	14 (3.2)	13 (4.3)	18 (6.7)	45 (4.4)	

TKM: Traditional Korean Medicine.

**Table 3:** Top five symptoms and diseases of outpatients using TKM.

NO	65 and Older		51 to 64		19 to 50	
	Symptoms of Diseases	Total *n* (%)	Symptoms of Diseases	Total *n* (%)	Symptoms of Diseases	Total *n* (%)
1	Osteoarthritis	166 (37.8)	Sprain	83 (27.6)	Disc related disease	78 (28.9)
2	Disc related disease	112 (25.5)	Osteoarthritis	74 (24.6)	Osteoarthritis	51 (18.9)
3	Back pain	37 (8.4)	Back pain	33 (11.0)	Back pain	30 (11.1)
4	Frozen shoulder shoulder pain	34 (7.7)	Frozen shoulder shoulder pain	29 (9.6)	Sprain	26 (9.6)
5	Sprain	26 (5.9)	Disc related disease	25 (8.3)	Frozen shoulder shoulder pain	23 (8.5)
Total	-	375 (85.3)		244 (81.1)	-	208 (77.0)

TKM: Traditional Korean Medicine.

**Table 4:** Treatments used for the top five symptoms in the older outpatient group (Unit: %).

	Conditions	Osteoarthritis	Disc Related Disease	Back Pain	Frozen Shoulder/Shoulder Pain	Sprain
Treatments						
Acupuncture		30.1	29.5	32.2	31.9	31.3
Moxibustion		19.3	17.0	22.0	17.2	18.4
Cupping		16.5	17.6	19.9	18.7	15.1
Pharmacopuncture		6.7	9.1	6.6	8.5	10.3
Herbal decoctions		5.7	3.2	2.4	3.3	4.6
Herbal extract		2.4	2.5	2.6	2.3	4.6
Physiotherapy		18.3	18.2	12.6	17.4	14.2
Chuna manual therapy		1.0	2.5	1.4	0.7	2.2

## Data Availability

The data will be made available upon reasonable request.

## Supplementary Materials

The following supporting information can be downloaded at: https://www.mdpi.com/article/10.3390/healthcare10081444/s1, Supplementary Materials S1: Utilization of Traditional Korean Medicine Services by the Older Population: A Cross-Sectional Study: A Survey.
