# Supplementary material for: Utilization of Traditional Korean Medicine Services by the Older Population: A Cross-Sectional Study
Source: Healthcare (Basel). 2022 Aug 1;10(8):1444. doi: 10.3390/healthcare10081444 (PMC9408479; doi:10.3390/healthcare10081444)
Supplement: Supplementary file 1 [file healthcare-10-01444-s001.zip › healthcare-1809588-supplementary.pdf]

Utilization of Traditional Korean Medicine Services by the Older Population: A Cross-Sectional Study: A Survey

Managing Department

Ministry of Health and Welfare

Agent in Charge of the Survey

NIKOM

[Objectives]

By collecting data to compared the older population (65 years and older) with the general population (19–64 years) to understand the current patterns in the use of TKM services by the older population. Using data from the 2017 National Survey of TKM Usage, we analyzed the main purpose and diseases or symptoms for TKM use, the reason for choosing TKM over other types of medicine, and the treatments provided.

[Targets]

The data compared and analyzed in this study were from a subset of the 1,010 patients in the 2017 National Survey for Usage and Consumption of Traditional Korean Medicine

[Items]

The survey items comprised two parts:

1. Basic characteristics: Gender, date of birth, married status, level of education, employment, income, state of health, type of health insurance

2. TKM services utilization: purposes of using the TKM services, reason for choosing the TKM services, symptoms of using the TKM services

[Instructions]

The Ministry of Health and Welfare intends to conduct a survey to examine the current status of the utilization of TKM services. The contents of this questionnaire will be used in statistical analysis for academic purposes only, and any personal information will remain strictly confidential other than for statistical analyses.

1. All responses expected to be answered within 1 year of TKM services experience.

2. All responses must reflect the current status as of the time of your completing of this questionnaire.

3. If you cannot find the option that best describes your response, you can fill in the blanks with relevant information in (other _______).

[PART 1. Basic Characteristics of Survey Group]

1. Gender

① Male ②Female

2. Date of birth

(Year__________) (Month__________)

3. What is your married status?

① Unmarried ② Married (bereaved, divorced and common-law included

4. What level of education have you completed?

① Primary or lower school graduate ② Middle school graduate ③ High school graduate ④University or higher school graduate

5. Are you currently employed?

① Yes ② No

6. What was your average monthly household income in the last year?

① Less than 1,500 USD ②1,500 USD less than 3,000 USD ③3,000 USD less than 4,500 USD ④4,500 USD less than 6,000 USD ⑤ No less than 6,000 USD

7. What is your current state of health?

① Good ② Bad ③ Average

8. What type of national health insurance do you have?

① Health insurance (district insurance) ② Health insurance (workplace insurance) ③ Medical care

[PART 2. TKM services utilization of Survey Group]

1. What is the purposes of using TKM services for the past year?

① Treating a disease ② Health promotion ③ Cosmetic treatment ④ Treatment for car accident

2. What is the main reason for choosing the TKM services?

① High effective ② Less side effects of surgery and examination ③ Less side effects ④ Low cost of treatment ⑤ to hear the detailed explanation ⑥ Treatment specific to the disease ⑦ Clinic's being closer ⑧ Good facilities ⑨ Famous and recommendation

3. Please check all the disease/symptoms of the TKM services you have used over the past year and respond to the treated intervention in the below.

① Acupuncture ② Moxibustion ③ Cupping ④ Pharmacopuncture ⑤ Herbal medicine preparations ⑥ Herbal decoctions ⑦ Physiotherapy management of the TKM ⑧ Tuina techniques ⑨ Other (_______)

**Disease classification**	**Diseases/Symptoms**		**Present=Yes Absent=No (circle)**	**Treated Intervention**
Musculoskeletal disease	1	Back pain	Yes No	
	2	Osteoarthritis	Yes No	
	3	Lumbar Spine	Yes No	
	4	Frozen shoulder (shoulder pain)	Yes No	
	5	Cervical spine	Yes No	
	6	Rheumatoid arthritis	Yes No	
Neurological disease	7	Disc related disease	Yes No	
	8	Sprain(shoulder, knee, wrist, ankle)	Yes No	
	9	Sprain(neck)	Yes No	
	10	Abdominal damage	Yes No	

Disease classification	Diseases/Symptoms		Present=Yes Absent=No (circle)	Treated Intervention
	11	Thoracic dislocation	Yes No	
	12	Femoral damage	Yes No	
Cerebrovascular disease	13	Headache	Yes No	
	14	Facial nerve palsy	Yes No	
	15	Partial paralysis	Yes No	
	16	Sleep disorder	Yes No	
	17	Parkinson's disease	Yes No	
Cardiovascular disease	18	Stroke of paralysis	Yes No	
	19	High blood pressure	Yes No	
	20	Ischemic heart disease	Yes No	
Cancer	21	Type of Cancer (______________)	Yes No	
Endocrine disease	22	Diabetes	Yes No	
	23	Thyroid disease	Yes No	
Digestive disease	24	Indigestion	Yes No	
	25	Gastritis, enteritis	Yes No	
	26	Bowel disorder	Yes No	

Disease classification	Diseases/Symptoms		Present=Yes Absent=No (circle)	Treated Intervention
Respiratory disease	27	Cold	Yes No	
	28	Rhinitis	Yes No	
	29	Bronchitis	Yes No	

Disease classification	Diseases/Symptoms		Present=Yes Absent=No (circle)	Treated Intervention
	30	Asthma	Yes No	
	31	Pneumonia	Yes No	
Mental disease	32	Dementia	Yes No	
	33	Mood disorder	Yes No	
	34	Anciety disorder	Yes No	
	35	Behavioral symdrome(ADHD, Tic)	Yes No	
Skin disease	36	Atopic dermatitis	Yes No	
	37	Rash, Urticaria	Yes No	
	38	Dry skin	Yes No	
	39	Hyperhidrosis	Yes No	
Obstetrics and gynecology disease	40	Menstrual disorder	Yes No	
	41	Infertility	Yes No	
	42	Urination disorder	Yes No	
	43	Prostate-related disease	Yes No	
Disease in eyes and ears	44	Tinnitus	Yes No	
	45	Otitis media	Yes No	
Others	46	Pelvic pain	Yes No	
	47	Dizziness	Yes No	
	48	Cough	Yes No	
	49	Hand tremor, Cramp	Yes No	

Disease classification	Diseases/Symptoms		Present=Yes Absent=No (circle)	Treated Intervention
	50	Skin Sensory disorder	Yes No	
	51	(Other________)	Yes No	
